# Intelligent Surveillance Robot with Obstacle Avoidance Capabilities Using Neural Network

**DOI:** 10.1155/2015/745823

**Published:** 2015-05-24

**Authors:** Widodo Budiharto

**Affiliations:** School of Computer Science, Bina Nusantara University, Jakarta, Indonesia

## Abstract

For specific purpose, vision-based surveillance robot that can be run autonomously and able to acquire images from its dynamic environment is very important, for example, in rescuing disaster victims in Indonesia. In this paper, we propose architecture for intelligent surveillance robot that is able to avoid obstacles using 3 ultrasonic distance sensors based on backpropagation neural network and a camera for face recognition. 2.4 GHz transmitter for transmitting video is used by the operator/user to direct the robot to the desired area. Results show the effectiveness of our method and we evaluate the performance of the system.

## 1. Introduction

Robotics has been a staple of advanced manufacturing for over half a century. As robots and their peripheral equipment become more sophisticated, reliable, and miniaturized, these systems are increasingly being utilized for entertainment, military, and surveillance purposes. A remote controlled surveillance robot is defined as any robot that is remotely controlled to capture images/video for specific purposes. Mobile robots that are controlled remotely have important rules in area of rescue and military.

A rescue robot is a kind of surveillance robot that has been designed for the purpose of rescuing people. Common situations that employ rescue robots are mining accidents, urban disasters, hostage situations, and explosions. Military robots are autonomous robots or remote-controlled devices designed for military applications. Such systems are currently being researched by a number of militaries. US Mechatronics has produced a working automated sentry gun and is currently developing it further for commercial and military use that can be operated remotely, and another very popular one is The Multi-Mission Unmanned Ground Vehicle, previously known as the Multifunction Utility/Logistics and Equipment vehicle (MULE) [1, 2].

Dealing with varied terrain places extra demands on the mobile robot's propulsion system, among other systems. Power management and new generation drive-train systems utilize advanced materials and highly efficient transmissions to obtain higher speed, accuracy, and durability to work in a wide range of environments. Enhanced power management comes through more advanced fuel cells and newly designed battery and charging systems.

Configuring a robot to ascend and descend obstacles in unstructured environments with ease is a design challenge and uses more power. The system must be able to overcome both regularly shaped obstacles such as stairs and those of an unspecified shape such as rocks, downed trees, and other miscellaneous objects. Engineers must consider the center of gravity, torque requirements to ascend inclines, mass, and payloads when designing mobile robotic systems for military purposes. In military applications, wearable robotics helps soldiers carry a heavy pack load. A robot acts like a pack mule, is fully autonomous, and carries a large amount of supplies [3].

There are many microcontrollers in the market consisting of various types of capability from basic input output to high end microcontroller. These various types of microcontroller are purpose-made for general application. In this research, we propose architecture for Raspberry pi based robot that can be controlled by neural network with the capabilities to avoid obstacles.

Over the last few years, a number of studies were reported concerning a neural network, bioinspired systems and computational intelligence, and how it has been applied to help mobile robots to improve their operational capabilities. Neural network deals with cognitive tasks such as learning, adaptation, and optimization. Recognition, learning, decision-making, and action constitute the principal navigation problems. Many researchers propose obstacle avoidance method for robotics such as [4]; they proposed obstacle avoidance method for two-wheeled mobile robot. Yue et al. [5] proposed a bioinspired collision detection with video sequences at 25 frames/s as a typical automotive scene that is used as input in Lobula Giant Movement Detector (LGMD) model as 150 × 100 greyscale bitmap images and operation of different genetic algorithms (GAs) are also investigated. Aihui and Deng also proposed NN operator-based robust nonlinear tracking control for a human multijoint arm-like manipulator with unknown time-varying delays by using robust right coprime factorization approach and a forward predictive operator [6]. That is, first, considering the uncertainties of dynamic model consist of measurement error and disturbances, a nonlinear feedback control scheme is designed to eliminate effect of uncertainties. Unfortunately, those systems tend to be very complex, need many tuning parameters because they are based on genetic algorithm (GA), use computationally intensive image processing which require long periods of time, and are not implemented in low cost surveillance robot. In our system, neural networks with their remarkable ability to derive meaning from complicated or imprecise data are suitable for mobile robot. We also propose camera system for detecting victims in the area of robot.

## 2. Design of Surveillance Robot with Obstacle Avoidances Capabilities

### 2.1. Architecture of Surveillance Robot

**Figure 1:**
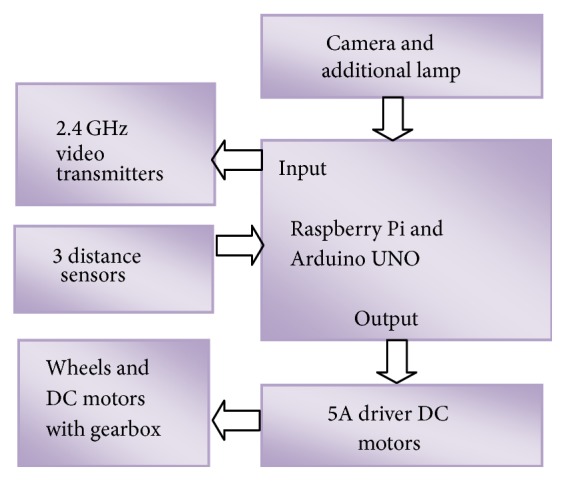
Architecture of remote controlled mobile robot.

Wheeled mobile robots originate from the autonomous mobile robot called “Yamabico” which has been studied for many years [7]. The robot used in this research is a mobile robot which is equipped with two actuator wheels and is considered as a system subject to nonholonomic constraints.  Figure 1 is a proposed block diagram of very low cost mobile robot for heavy load that consists of Raspberry pi, distance sensors, and Arduino [8], 5A driver DC motors, and DC motors with wheels. For the driver of DC motor, we use MOSFET with the low resistance of the drain-source. The output of AV port of Raspberry pi connected to 2.4 GHz transmitter for video transmission. Additional lamp is used for lighting the area in front of robot; this is very useful for face recognition.

**Figure 2:**
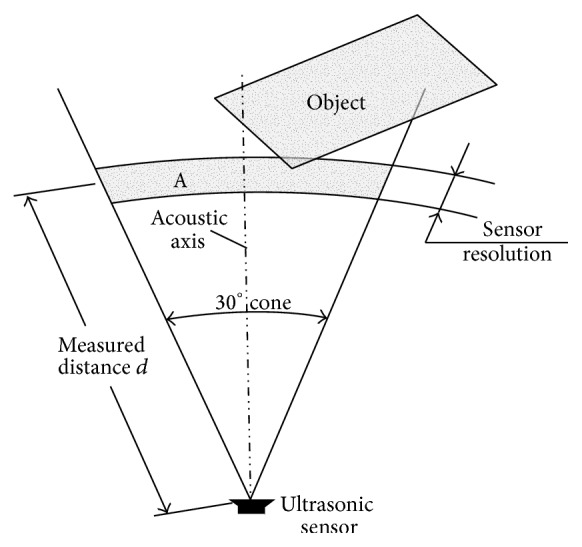
Two-dimensional projection from conical fields of ultrasonic sensor. The distance measurement *d* indicates the existence of an object in the area [10].

At previous work [9], we prove that our method using 3 ultrasonic distance sensors is enough for detecting obstacle, so we implement that method for this research. Ultrasonic sensors work at a frequency of 40 KHz and have a deviation angle maximum of about 30°, so usually robots need more than one sensor to be able to measure the distance of an obstacle in its vicinity (Figure 2). The main weakness of this type of sensor is the interference between different sensors and the limited ability to identify the obstacle. The advantage of this type of sensor is that it is usually able to detect the obstacle at a distance ≥3 cm, something a vision sensor is not able to do.

Sensor detects objects by emitting a short ultrasonic burst and then “listening” for the echo. Under control of a host microcontroller (trigger pulse), the sensor emits a short 40 kHz (ultrasonic) burst. This burst travels through the air, hits an object, and then bounces back to the sensor. The PING))) sensor provides an output pulse to the host that will terminate when the echo is detected; hence the width of this pulse corresponds to the distance to the target.

### 2.2. Neural Network Architecture

Many techniques have been developed to carry out obstacles avoidance efficiently by using recent sensor data [11]. In our method, we make a simple decision to check whether there is obstacle or not by use 2 variables, far and near. Far if there is no obstacle (>60 cm), and near if the distance between robot and obstacle is <60 cm.

Backpropagation is an algorithm in neural network that can be used to train a neural network. Training a neural network is the process of finding a set of weights and bias values so that, for a given set of inputs, the outputs produced by the neural network are very close to some known target values. Gradients are values that reflect the difference between a neural network's computed output values and the desired target values. As it turns out, gradients use the calculus derivative of the associated activation function. The gradients of the output nodes must be computed before the gradients of the hidden layer nodes, or in other words, in the opposite direction of the feedforward mechanism.

Supervised learning which incorporates an external teacher, so that each output unit is told what its desired response to input signals is used in our system. Backpropagation is basically a gradient descent process, with each change in the weights of the network bringing the network closer to a minimum error represented in a multidimensional weight space. Gradient descent does have its problems; however, in backpropagation, these problems manifest themselves as the time taken to reach a minimum and the occurrence of local minima. Let *x* be an input; *w* is weight and the output is *y*_*k*_. The algorithm for backpropagation is shown below.

**Algorithm 1 (backpropagation).**

 Initialize each *w*_*i*_ to some small random value
 While not reach termination condition
 Do
 For each training example 〈(*x*_1_,…, *x*_*n*_), *t*〉 Do
   Input the instance (*x*_1_,…, *x*_*n*_) to the network and compute the
   network outputs *y*_*k*_
 For each output unit *k*
  (1) $$\begin{array}{c} \delta_{k}=y_{k}\left(\;1-y_{k}\;\right)\left(\;t_{k}-y_{k}\;\right) \end{array}$$
 For each hidden unit *h*
  (2) $$\begin{array}{c} \delta_{h}=y_{h}\left(\;1-y_{h}\;\right)\overset{}{\underset{k}{\sum }}w_{h,k}\delta_{k} \end{array}$$
 For each network weight *w*_*i*,*j*_ Do
  (3) $$\begin{array}{c} w_{i,j}=w_{i,j}+\Delta w_{i,j},\;where\;\;\Delta w_{i,j}=\eta \delta_{j}x_{i,j} \end{array}$$
 End Do

**Figure 3:**
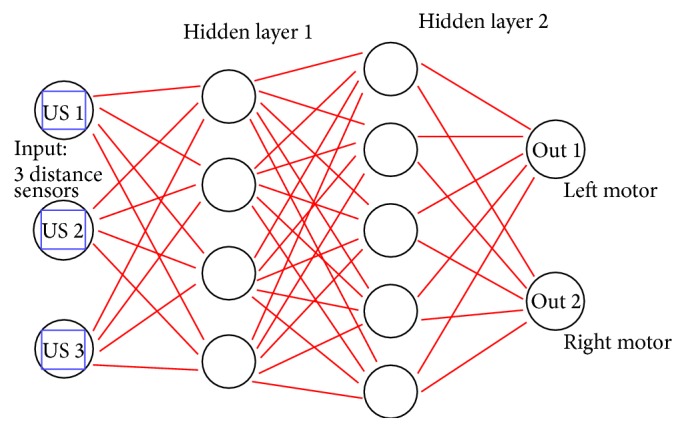
Three input sensors trained using neural network.

Figure 3 is our multilayer perceptron architecture with 3 inputs, 2 hidden layers, and 2 outputs to control the motors. We use 60 training data, learning rate = 0.05, and momentum 0.01 for this experiment.

**Figure 4:**
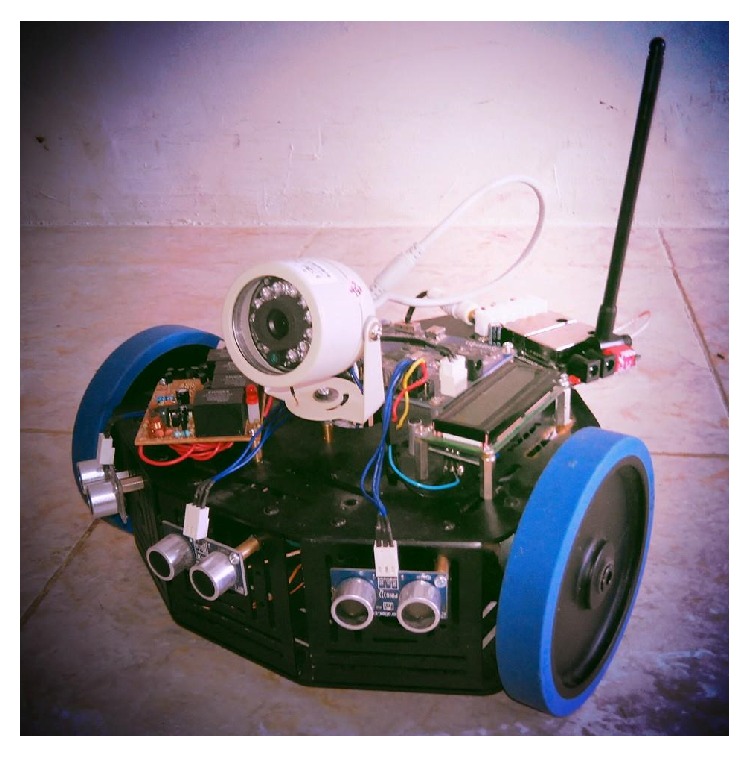
Three distance sensors for obstacles avoidance and camera transmitted using 2.4 GHz transmitter.

The prototype of surveillance robot is shown in Figure 4.

### 2.3. Algorithm for Surveillance Robot

We want to make sure that our system can be applied in real word such as production robot system [12]. So, we improve the mobile robot developed before [13]; we have developed algorithms and programs consisting of 3 main modules, namely, the ActivateCamera(), ObstacleNeuralNetwork(), and the maneuvering method. The algorithm is shown in Algorithm 2.

**Algorithm 2 (remote controlled surveillance robot).**

 Declare variables
 Declare functions
 Set all motors off
 Initialize the sensors
 Far is greater than 60 cm
 Near is below 60 cm
 Do
   Call ActivateCamera()
   Call ObstacleNeuralNetwork()
   Call ManeuveringMethod()
 If front distance is far then
   Call forward
 End if
 Function ManeuveringMethod
 If front distance is near then
   Call backward
 End if
 If front distance is far and front right is far and front left is near then
   Call turn_right
 End if
 If front distance is far and front left is far and front right is near then
   Call turn_left
 End if
 If front distance is near and front right is near and front left is near then
   Call backward
 End if
 End function

## 3. Experimental Result

Our proposed method for surveillance robot named RoboExplorer ver. 1.0 has been successfully implemented and it has shown a good performance in our laboratory. The drive wheel as well as the passive wheels is equipped with shaft encoders used for odometry measurement. Three ultrasonic sensors succeeded to detect and measure the distance of obstacle continuously as shown in Table 1.

**Figure 5:**
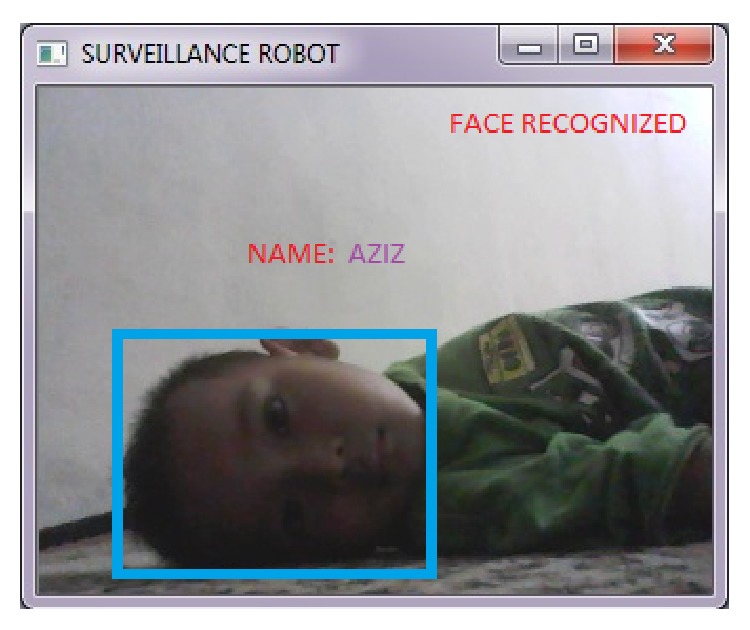
Face recognition system using OpenCV to recognize the victims.

We have implemented the algorithm described in the above sections in our system using C++ and Raspberry pi. The result of video streaming with face recognition capabilities using OpenCV and transmitted using 2.4 GHz is shown in Figure 5.

## 4. Conclusion

This paper presents a new method of vision-based surveillance robot with obstacles avoidance capabilities for general purpose robot in indoor environments. Algorithms of neural network for obstacle avoidance were implemented in the robot. Experimental results with various positions of obstacle show the ability of robot to avoid it and have shown a good performance. The robot is also able to recognize victims in front of the robot. The sensor system is very cheap because it only uses 3 distance sensors. For future work, we will improve this system for swarm robotics system.

## Figures and Tables

**Table 1:** Result of obstacle avoidance using NN.

Number	Action	
1	Avoiding obstacle in front of robot	Success
2	Avoiding obstacle at the left of robot	Success
3	Avoiding obstacle at the right of robot	Success
